# Assessing lead exposure in U.S. pregnant women using biological and residential measurements

**DOI:** 10.1016/j.scitotenv.2023.167135

**Published:** 2023-09-21

**Authors:** Lindsay W. Stanek, Nicholas Grokhowsky, Barbara J. George, Kent W. Thomas

**Affiliations:** a U.S. Environmental Protection Agency (EPA), Office of Research and Development (ORD), Research Triangle Park, NC, USA; b Formerly of Oak Ridge Institute for Science and Education, Research Triangle Park, NC, USA

**Keywords:** Blood lead, Urine lead, Surface wipe, In utero, Exposure determinants

## Abstract

There is strong scientific evidence for multiple pathways of human exposure to lead (Pb) in residential settings, particularly for young children; however, less is known about maternal exposure during pregnancy and children’s exposure during early lifestages. A robust, multi-faceted secondary analysis was conducted using data collected by the National Institute of Child Health and Human Development in the 2009-2014 National Children’s Study Vanguard Studies. Descriptive statistics summarized Pb concentrations of maternal blood, maternal urine, and house dust vacuum samples collected during pregnancy and residence surface wipes collected both during pregnancy and six months post-partum. The maternal blood Pb level geometric mean was 0.44 μg/dL (*n* = 426), with no women having values ≥ 5 μg/dL; creatinine-adjusted maternal urinary Pb geometric mean was 0.43 μg/g (*n* = 366). These blood and urine concentrations are similar to those observed for females in the general U.S. population in the National Health and Nutrition Examination Survey 2010–2011 cycle. A modest correlation between maternal blood Pb and surface wipe measurements during pregnancy was observed (Spearman *r* = 0.35, *p* < 0.0001). Surface wipe Pb loadings obtained in mother’s homes during pregnancy (*n* = 640) and from areas where children spent the most time at roughly 6 months of age (*n* = 99) ranged from 0.02 to 71.8 ng/cm^2^, with geometric means of 0.47 and 0.49 ng/cm^2^, respectively, which were relatively low compared to other national studies. Survey responses of demographic, lifestyle, and residence characteristics were assessed for associations with blood concentration and surface wipe loading. Demographic (e.g., race/ethnicity, income, education, marital status) and housing characteristics (e.g., year home built, paint condition, own or rent home, attached garage) were associated with both maternal blood and surface wipe loadings during pregnancy. The availability of residential environmental media and extensive survey data provided enhanced understanding of Pb exposure during pregnancy and early life.

## GRAPHICAL ABSTRACT

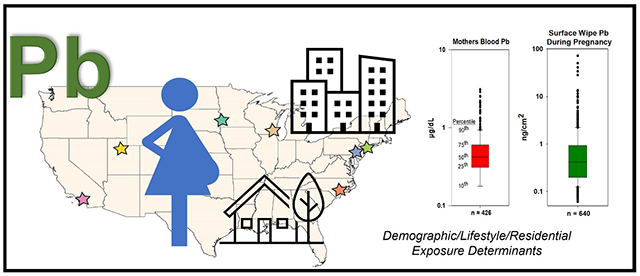

## 1. Introduction

Lead (Pb) is a ubiquitous environmental pollutant that remains a potential hazard for human exposure in the United States. Significant progress has been made to reduce Pb concentrations at the source (e.g., removal of Pb from gasoline, elimination of Pb from paint); however, decades of use resulted in Pb as a legacy contaminant that is still detected in the environment. There are well documented health effects of Pb in young children, where it can affect neurodevelopment and cognitive function, even at blood lead levels (BLL) well below 10 μg/dL (Arnold and Liu, 2020; Jedrychowski et al., 2009). The large body of science suggests that no threshold exists below which children’s health is unaffected (ATSDR, 2020).

Pregnant women and the fetus may also be impacted by prenatal Pb exposure, as Pb can be released from maternal trabecular and cortical bone stores during fetal skeleton development (Gulson et al., 2003; Miranda et al., 2010). Evidence is sparse at low maternal BLLs for physiological changes or birth outcomes, albeit maternal hypertension (Yazbeck et al., 2009) and preterm birth have been reported (Jelliffe-Pawlowski et al., 2006; Vigeh et al., 2011). A recent analysis of the National Children’s Study (NCS) Vanguard Studies demonstrated decreased gestational age, birthweight, birth length, and head circumference for female infants associated with maternal BLL (Shih et al., 2021). Mixed results have been observed for in utero exposure studies that attempt to link maternal BLL (whether in blood or cord blood) and offspring neurodevelopment effects (Jedrychowski et al., 2009; Polanska et al., 2018; Taylor et al., 2017). Widespread screening for BLLs in pregnant women does not exist in the U.S.; however, for those who are tested, the Centers for Disease Control and Prevention (CDC) recommends follow-up actions with BLL ≥ 5 μg/dL (CDC, 2010).

Residential Pb sources for pregnant women are also likely to be important potential exposure pathways for their young children during infancy and toddlerhood, critical periods of development that coincide with hand-to-mouth activity and oral sensory seeking behavior. Associations between children’s BLL and house dust as a main source of exposure to Pb are well established (Adgate et al., 1995; Becker et al., 2022; Dong et al., 2020; Lanphear et al., 1998). Limited studies in pregnant women and measured environmental samples found dissimilar results: no relationship between BLL and house dust Pb concentrations in a Japanese cohort (Ohtsu et al., 2019) and a positive association with soil Pb in a cluster analysis in Durham, NC (King et al., 2015).

Adverse health impacts resulting from maternal and children’s exposures to Pb can also be considered in a total environmental framework perspective (Tulve et al., 2016). Demographic characteristics including minority race/ethnicity, lower income, and geographic location proximate to industry or contaminated land and water, often associated with exposures to Pb, may also be associated with co-exposures to other pollutants (Whitehead and Buchanan, 2019). Other social determinants of health, such as lower access to health care, reduced economic stability, and limited access to green space may be important for pregnant women (Sharma et al., 2022; Wang et al., 2020; Zhan et al., 2020). Cumulative impacts of chemical and non-chemical exposures may be particularly important in environmental justice communities (Peters et al., 2019; Prochaska et al., 2014). Social determinants of health are comingled and often difficult to tease apart, but research that widely examines the social and chemical environment can improve our understanding of community vulnerability to Pb exposures.

Given the limited data available to evaluate Pb contamination in the home environment and associated concurrent maternal biomarker measures of exposure, we utilized data from the NCS Vanguard Studies to better understand these source-to-exposure relationships. Through a secondary data analysis, distributions of Pb concentrations in maternal blood and urine and in residential samples (i.e., home surface wipes and vacuum house dust) were characterized and their associations examined. Potential determinants of Pb exposure were assessed using NCS sociodemographic, lifestyle, occupational, and residential characteristic survey responses in one-way analysis of variance (ANOVA) and multivariable models, specifically multiple regression, for BLL and surface wipe loadings.

## 2. Materials and methods

### 2.1. Study population

We performed secondary data analyses using survey response and chemical measurement data collected between 2009 and 2014 in the National Institute of Child Health and Human Development (NICHD) NCS Vanguard Studies. The NCS Main Study was a proposed nationally representative longitudinal birth cohort study intended to assess relationships between environmental and other exposures and children’s health in the United States (Hirschfeld et al., 2011; Montaquila et al., 2010; Mortensen and Hirschfeld, 2012). While the full NCS Main Study was never implemented, a pilot study called the Initial Vanguard Study (IVS) was implemented in 2009–2010 to test recruitment methods and protocols for the anticipated Main Study (Baker et al., 2014). Over 1300 women, including 970 who were pregnant, were enrolled in the IVS from six counties and one group of four contiguous rural counties in the United States. Demographic information was obtained for all IVS participants; however, data collected by questionnaires, observation survey instruments, and as biological and environmental samples were obtained for subsets of participants. Specifically, lifestyle, residence, occupational, and other types of survey data were available from NCS questionnaire and observational survey instruments. Biological and environmental samples were collected for chemical analyses to assess exposure assessment procedures (Abdul-Rahman et al., 2016; Boyle et al., 2015; Needham et al., 2005). Pregnant IVS participants provided blood samples (*n* = 432), urine samples (*n* = 366), and/or residence surface wipe samples collected during pregnancy (*n* = 640) and at six months post-partum (*n* = 99) that were analyzed for elements, including Pb. Additionally, in the NCS Alternative Recruitment Substudies (ARS) implemented between 2010 and 2014, Pb measurements were available from vacuum house dust samples collected from the homes of pregnant women (*n* = 208) enrolled in 20 U.S. counties (Blaisdell et al., 2016; Hale et al., 2016; McGovern et al., 2016).

### 2.2. Data acquisition

We performed data analyses in two phases. Initial data analyses were performed within the NCS Vanguard Data and Sample Archive Access System (NICHD, 2016) in February 2020 prior to the closing of the Archive system in April 2020. These initial time-limited analyses focused on assessing geographical differences in Pb measurements across the seven IVS locations. Following transfer of the NCS Archive to NICHD’s Data and Specimen Hub (DASH) (Moye, 2020), in the second phase of the project we retrieved biological and environmental measurement data sets for descriptive and multivariable Pb analyses. We also retrieved NCS survey response data, specifically, an enrollee demographics dataset, “T1-First” and “T3-First” prenatal questionnaire datasets, a structures dataset reporting study technician observations outside residences, and a dwelling dataset reporting study technician observations inside residences. The T1-First questionnaire was administered for participants recruited during their first trimester of pregnancy while the T3-First questionnaire was administered for participants enrolled in their third trimester. Geographic location information was not provided in DASH datasets, preventing subsequent inclusion in multivariable analyses.

### 2.3. Summary statistics and media correlation analyses

Our primary data analysis and reporting were focused on maternal blood and residence surface wipe samples collected during participants’ pregnancy. These are key data for understanding both pregnant women’s exposures and young children’s potential exposures to surface-available Pb. Data sources and criteria for selection of participants to include in the analyses are described in Table S-1. We note that three participants in the DASH blood data set had two observations each; all six data points were removed prior to analyses since the rationale for two differing measurements could not be resolved.

We examined participant characteristics for selected demographic, lifestyle, and residential variables for 426 participants with usable BLLs and 640 participants with surface wipe results, both collected during pregnancy. We compared these two groups noting that 357 participants provided both blood and surface wipe samples. While our primary analyses focused on blood and prenatal surface wipe Pb measurements, we generated summary statistics for multiple available media including blood, urine, surface wipe, and vacuum dust. Statistics for BLLs and urinary Pb included percent above the limits of detection (LOD) of 0.25 μg/dL and 0.11 μg/g, respectively, geometric means (GM) and their 95 % confidence intervals (CI), and selected percentiles of the distributions. Some measurements for BLLs (14 %) and urinary Pb (3 %) were < LOD, so we used maximum likelihood estimation for censored data in the R package *EnvStats* version 2.3.1 to calculate their geometric means and 95 % CIs (Millard, 2018). Spearman correlation coefficients were calculated to assess relationships between participant’s blood and surface wipe (*n* = 357), blood and urine (*n* = 179), and urine and surface wipe (*n* = 329) measurements using R version 3.6.2 (R Core Team, 2014). Spearman correlation analyses used LOD/√2 substitution for censored measurements < LOD. All surface wipe and vacuum dust measurements were > LOD.

### 2.4. Exposure determinants assessment

In the initial analysis phase working in the NCS Data Archive, the Pb measurements were organized into seven geographic units (six counties and one county group). A total of 421 unique participants had both BLLs and geographic location data and 640 participants had prenatal surface wipe and geographic location data. We calculated Pb geometric means for each geographic unit and used one-way ANOVA in R version 1.3.9 to test for differences across the geographic units using the natural log of the Pb measurements. Our analyses in this initial phase used LOD/√2 substitution for censored measurements < LOD.

In the second phase of analyses using NCS data retrieved from DASH, we assessed determinants potentially associated with lower or higher Pb exposures. Specifically, we examined blood and surface wipe measurement relationships with 43 survey variables (including demographic, lifestyle, occupation, time/activity, and residence information) and two sample-associated variables (blood collection trimester and serum cotinine concentrations) using one-way ANOVA and multivariable (i.e., multiple regression) analysis. ANOVA exposure determinant analyses used LOD/√2 substitution for censored BLL < LOD. Multivariable modeling used both maximum likelihood estimation for censored data and LOD/√2 substitution for censored BLL < LOD. Urine, post-partum surface wipe, and vacuum dust measurements were not included in these exposure determinant assessments.

Our analyses were complicated by survey response data availability and needed recodes. For some variables there were no responses for subsets of participants because they were not administered a particular survey instrument. In other cases, response categories with low response frequencies were combined with responses for another category to form larger frequency groups. For example, the maternal education variable included response categories for associates degree, bachelor’s degree, or advanced degree with 7, 22, and 11 participants with BLLs, respectively. These categories were combined to form one college degree category with 40 participants. In several cases, we derived analysis variables from two or more other variables included in NCS survey datasets. These derived variables included tobacco use, perceived stress based on the 10-item Perceived Stress Scale (PSS-10) (Cohen et al., 1983; Cohen and Williamson, 1988), household person density, how long participants resided in their home, parity, and maternal BMI (Table S-2). In cases where more than one type of missing category was included, all participants with missing data were combined into one category. Any survey response or sample-derived category with fewer than 10 observations was excluded from analysis. We calculated Pb geometric means for each category and tested differences among them using one-way ANOVA (R version 3.6.2).

We fitted a main effects multiple regression BLL model for fifteen variables that had *p* ≤ 0.10 in the one-way ANOVA models. This main effects model used data for 283 of the 426 participants in the BLL data set with complete data for all variables; 48 of these participants included in the model had BLL measurement data <LOD. A second BLL model with interactions was fitted for the 283 participants using forward and backward selection. This full model gave an improved fit relative to the main effects model based on AIC. Both multiple regression models were fitted using the SAS LIFEREG procedure in a Tobit analysis using maximum likelihood estimation for censored data (SAS/STAT, 2019). Main and full fixed effects models were also fitted for comparison using the SAS MIXED procedure with LOD/√2 substitution for the 48 observations with left-censored BLL measurements. Use of substitution for censored outcome variables is not a statistical best practice but remains a conventional approach despite arguments against it (George et al., 2021).

We also fitted a main effects multiple regression surface wipe Pb model for fourteen variables that had *p* ≤ 0.001 in the one-way ANOVA models. This main effects model used data for 450 of the 640 participants in the surface wipe Pb data set with complete data for all variables; none of the participants had left-censored surface wipe Pb measurement data. A second model with interactions was fitted for 475 participants using forward and backward selection and found significant joint associations for eleven of the fourteen variables. Three variables not found to be significant in this second model were omitted (i.e., perceived neighborhood safety, yard cleanliness, and interior carpet quality). The surface wipe multiple regression models were fitted using the SAS MIXED procedure for fixed effects using maximum likelihood estimation since all measurements were > LOD (SAS/STAT, 2019).

There was considerable overlap in participants with data included in the BLL and surface wipe models. Out of 709 total participants, 357 participants had data for both, 69 had data for blood only, and 283 had data for surface wipe only. Most of the data used in the BLL models was from participants whose data was also used in the surface wipe Pb models. Specifically, the BLL models used data for 283 participants, and 246 of these participants were also included in the surface wipe Pb models. Approximately half of the data used in the main effects surface wipe Pb model was from participants whose data was also used in the BLL models; the surface wipe model used data for 450 participants with 246 of these participants also reflected in the BLL models.

### 2.5. Quality control/quality assurance

Second-person verification was performed for the creation of analytical data files for Pb measurements, selected summary statistics calculations, Spearman correlation coefficient calculations, and creation of recoded survey variable files for determinants assessment. One-way ANOVA calculations performed using R were confirmed by a second person for a subset of variables using SAS, except for the geographical differences assessment for which second-person verification was performed using R. Graphical assessments and statistical tests from the R *EnvStats* package goodness-of-fit functions were used to understand the distributional form of the Pb measurement data sets to inform statistical analysis decisions. Procedures and assessments were subjected to a quality assurance audit.

## 3. Results and discussion

### 3.1. Descriptive statistics

Characteristics of the NCS participants included in the analyses are shown in Table 1. Overall, there were no major differences between the participants providing blood samples and those providing surface wipe samples, and 357 individuals provided both. Most participants were non-Hispanic White and there were fairly even distributions of family income and home ages across the respective categories. A notable difference between the two groups was that all but one of the participants providing blood samples responded to the first trimester version of the prenatal questionnaire (T1-First), whereas many surface wipes were obtained for participants who completed the third trimester version of the prenatal questionnaire (T3-First). Consequently, pre-pregnancy tobacco and alcohol use information was not available for over 22 % of participants who provided surface wipe samples (compared with only 6 % for those providing blood) because the third trimester version of the questionnaire did not collect that information. Five percent of the participants blood samples had no corresponding questionnaire data.

**Fig. 1.**
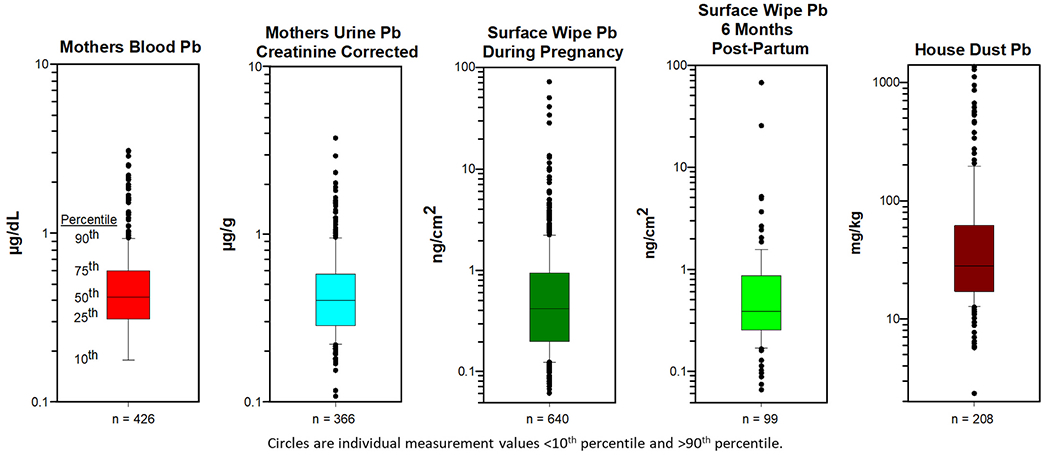
Boxplots showing distribution quantiles of Pb measurements in several media for NCS participants where LOD/√2 substitution was used for blood and urine measurements <LOD. All surface wipe and house dust measurements were >LOD.

Distribution quantiles of Pb measurements for NCS blood, urine, surface wipes, and vacuum dust are presented in boxplots in Fig. 1 and Table S-3. BLLs were generally low (geometric mean = 0.44 μg/dL), with no participants having BLL ≥ 5 μg/dL (Table S-3). The geometric mean BLL for pregnant women in NHANES from 2011 to 2016 was slightly higher in comparison at 0.48 μg/dL with 2.8 % having BLL ≥ 5 μg/dL (Ettinger et al., 2020). For non-pregnant women in NHANES, the geometric mean BLL was even higher at 0.61 μg/dL than for the pregnant women (Ettinger et al., 2020) (Table S-4). When comparing across NCS IVS locations, maternal BLLs were significantly different, with Queens County, NY having the highest geometric mean value (0.72 μg/dL) (Fig. S-1). Participants residing in central U.S. locations (i.e., South Dakota/Minnesota, Wisconsin, Utah) had the lowest (< 0.40 μg/dL) geometric mean BLLs (Table S-5 and Fig. S-1). A one-time BLL measurement reflects both recent exposure and body burden from bone stores, but in pregnant women it may also be associated more directly to remobilization from bone with the increased calcium demand that accompanies a developing fetus (Gulson et al., 1997). However, a study of pregnant women in Durham, NC concluded that historic exposures, particularly in older mothers (i.e., >30 years old), were more likely the source of maternal blood Pb (Miranda et al., 2010). No significant association between BLL and age was observed in the NCS; however, it is difficult to attribute Pb to current or historical exposure in the NCS cohort with its relatively low BLLs.

Children’s exposure to Pb prenatally and in early childhood is a concern shared around the world. Consequently, Pb exposure for pregnant women has been widely studied, most often assessed through blood Pb measurements. A comparison of results for pregnant women in this U.S. NCS cohort to those from studies conducted in 11 countries in North America, the Caribbean, South America, Europe, Asia, and Oceana found that, overall, the NCS geometric mean and median BLL measurements were lower (Table S-6). The geometric mean BLL in the NCS cohort (0.44 μg/dL) was lower than the geometric mean BLLs measured in contemporaneous North American studies in Canada (0.57–0.62 μg/dL) and Mexico (3.0–3.1 μg/dL), noting that the Mexico results were substantially higher than those from both the U.S. and Canada (Arbuckle et al., 2016; Renzetti et al., 2017). The median BLL (0.42 μg/dL) in the NCS cohort was also substantially lower than median BLLs measured in Grenada (1.09 μg/dL), St. Vincent and the Grenadines (2.07 μg/dL), and in a more recent study in Brazil (0.9 μg/dL) (Forde et al., 2014; Bah et al., 2023). Median BLL measurement results in studies in the European nations of Norway (0.82 μg/dL), Poland (1 μg/dL), and Spain (1–1.2 μg/dL) were also found to be higher than the NCS median BLL; and the geometric mean results for an earlier Spain cohort measured (1.98 μg/dL) was substantially higher than the NCS geometric mean (Bocca et al., 2019; Caspersen et al., 2019; Kot et al., 2021; García-Esquinas et al., 2013). The geometric mean in a large birth cohort study in Japan (0.64 μg/dL) was slightly higher than the geometric mean measured in the NCS cohort (Nakayama et al., 2019). The median Pb level for a large contemporaneous study in China (0.19 μg/dL) was substantially lower than the median BLLs from all the other countries in this comparison, including the U.S.; however, the study in China measured Pb in serum, unlike the other studies, possibly limiting the direct comparability to measurements made using whole blood samples (Liu et al., 2017). There is one exception in this comparison to NCS reporting the lowest mean and median BLLs in whole blood measurements. The median BLL from the study in Australia (0.37 μg/dL) was lower than the median BLLs in most other countries, including NCS in the U.S. (Hinwood et al., 2013).

An assessment of the relatively low U.S. NCS geometric mean and median BLLs against those from the other countries’ studies in our comparison did not reveal a potential explanation for the U.S.’s ranking. We looked at starting and ending years and also at mean maternal age given that BLL levels tend to be higher with higher maternal age. The starting and ending years for NCS (2009 and 2010, respectively) are approximately mid-range of the other studies (2002–2022). The mean maternal age for NCS (29.2 years) is just under mid-range of the ages in this comparison (26.5–34.0 years). We find it reassuring, however, that the NCS geometric mean (0.44 μg/dL) is consistent with that in a contemporaneous U.S. NHANES cohort that is nationally representative (0.48 μg/dL) (Table S-4).

Urinary lead measurements in pregnant women provide another dimension for understanding children’s prenatal exposure to lead. A comparison of results for pregnant women in this U.S. NCS cohort in 2009–2010 to urinary Pb measurements from other studies found mixed results. For creatinine-adjusted urinary Pb levels, a direct comparison to NHANES for pregnant women or women of childbearing age was not available, as the data are not grouped similarly (CDC, 2022). The NCS geometric mean of 0.43 μg/g was, however, quite similar to geometric means for all NHANES participants 20+ years and females aged 6+ years, particularly for 2011–2012 (Table S-7). The creatinine-adjusted urinary Pb concentrations were the largest for the 2009–2010 NHANES subsets, including at the upper end of the distribution (i.e., 90th percentile values above 1.20 μg/g) (Table S-7). Other studies report data for pregnant women or women of childbearing age, including studies in the U.S., Sweden, and Spain. The geometric means of creatinine-adjusted urinary Pb concentrations of pregnant women in Houston, TX and Boston, MA were lower than those observed in the NCS at 0.17 μg/g and 0.27 μg/g, respectively (Han et al., 2020; Kim et al., 2018). In a group of women aged 21–64 years old from Sweden, 24-h or overnight sample creatinine adjusted urinary Pb geometric means of 0.34 and 0.30 μg/g, respectively, were lower than the NCS’s 0.43 μg/g (Sallsten et al., 2022). In contrast, pregnant women from Spain had higher median creatinine-adjusted urinary Pb concentrations that ranged between 1.2 and 1.4 μg/g depending upon the trimester the sample was collected (Bocca et al., 2019).

For surface wipes, geometric mean Pb loadings were similar between the NCS samples taken during pregnancy (0.47 ng/cm^2^) and 6-months postpartum (0.49 ng/cm^2^) (Fig. 1). The 95th percentile value for surface wipes collected during pregnancy was 3.6 ng/cm^2^, which is below the current EPA dust Pb clearance level of 10.8 ng/cm^2^ (i.e., 10 μg/ft^2^); however, the top 1.2 % of homes exceeded that level (EPA, 2021). When comparing across NCS IVS locations, surface loadings were significantly different, with Queens County, NY having the highest geometric mean value (1.21 ng/cm^2^) and Duplin County, NC having the second highest value (0.82 ng/cm^2^) (Table S-5). Women residing in central U.S. locations (i.e., South Dakota/Minnesota, Wisconsin) and in Orange County, CA had geometric mean surface loadings <0.40 ng/cm^2^. Surface wipe loadings for NCS participants were generally lower than Pb loadings observed in either the American Healthy Homes Survey (AHHS) I or II from 2005 to 2006 and 2018–2019, respectively (HUD, 2021). However, the median NCS Pb wipe loading was greater than the median for AHHS II while the 90th percentile value was greater in AHHS II, reflecting pronounced skewness in the AHHS II measurements (Table S-8). The observed Pb in surface wipes may, for example, be due to deteriorating indoor paint or renovation activities that disturb Pb-based paint. Transfer of outdoor soils to the interior of a residence through human activity or building ventilation and occupational-derived exposures that constitute “take-home” Pb could also be potential sources for indoor surface contamination.

### 3.2. Correlation assessments

**Fig. 2.**
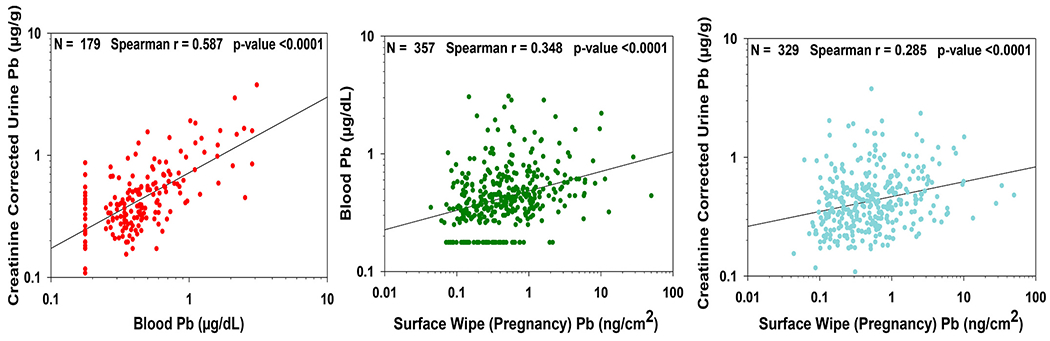
Correlation coefficients for Pb measurements in selected media where LOD/√2 substitution was used for blood and urine measurements <LOD. All surface wipe measurements were >LOD.

Spearman correlation coefficients for NCS Pb biomarkers and surface wipes were all statistically significant, with the strongest result between BLLs and creatinine corrected urinary Pb (Fig. 2). This correlation is not surprising, as urinary Pb generally reflects the concentration of Pb in the blood when samples are collected at roughly the same time (Sallsten et al., 2022). Diamond et al. (2019) recently found a 1:1 relationship between glomerular filtration rate (GFR) and clearance of blood Pb in NHANES data, which they attributed to a strong association between plasma Pb and BLL. In contrast to Sallsten et al. (2022), a study in Australian pregnant women found no relationships between urinary Pb and BLLs obtained in the third trimester (Hinwood et al., 2013).

There are very few studies in the literature that have obtained concurrent blood, urine, and wipe samples from pregnant women. One study conducted in Japan with pregnant women did not find statistically significant correlations between BLL and vacuum dust Pb concentrations (Ohtsu et al., 2019). Although not indoor dust, King et al. (2015) found soil Pb levels significantly correlated with the BLLs of pregnant women in Durham, NC. A seminal study of a pooled analysis of young children’s BLL and environmental Pb variables (i.e., dust, soil, paint, and water) showed that floor dust Pb loading was a significant predictor of BLL, even at loadings below 10 μg/ft^2^ (Lanphear et al., 1998). The positive and significant correlation between blood and surface wipes in NCS is noteworthy, as Pb exposure can originate from numerous multimedia sources that were not quantitatively measured in the NCS. Drinking water and dietary pathways can be important contributors to Pb exposure at individual and community scales. Other personal behaviors in pregnant women may be risk factors for elevated BLL, including the eating disorder pica, use of alternative remedies or cosmetics, and use of traditional Pb-glazed pottery (CDC, 2010). While a direct physical basis for increased BLL with exposure to higher Pb surface loadings is plausible, a correlation assessment cannot rule out factors that might independently lead to both higher BLL and surface loadings based on different sources and exposure pathways.

### 3.3. Regression analysis

Information for a large number of demographic, behavior and lifestyle, and housing variables was collected in the NCS IVS; many were examined for their associations with BLL and Pb surface loading. A majority exhibited significant associations with surface loading, while a smaller number were significant for BLL. For the one-way ANOVA analysis, the variables of race, education, year home built, and exterior siding condition had the strongest results (*p*-value ≤0.0001) for both blood and surface wipes (Table 2). Having lower educational attainment (GED or less than high school) and poor exterior siding condition were each associated with higher geometric mean BLL and surface loadings. For race/ethnicity, the pattern differs between blood and surface wipes, with the highest geometric mean BLLs observed for Asian and then Hispanic women (0.99 and 0.58 μg/dL, respectively). However, the number of participants classified as Asian was the lowest of the race categories (*n* = 20). The greatest geometric mean Pb loadings measured in surface wipes were observed for Black/African American and Hispanic women (0.90 and 0.88 ng/cm^2^, respectively). For year home built, there was a clear pattern for geometric mean surface loadings with the lowest loading for 2001-present with a gradual increase with housing age; the highest loading was in homes built prior to 1940 (1.13 ng/cm^2^). When looking at BLL, the largest geometric mean value was in the don’t know/missing category (0.58 μg/dL), followed by 1940 or before (0.50 μg/dL) and 1941 to 1960 (0.48 μg/dL). A large proportion of women (18–20 %) did not know the age of the building they lived in, possibly because the homes were older and/or the women were renters.

There were additional sociodemographic and residential variables with statistically significant associations with surface loadings in the one-way ANOVA regression models. For example, lower household income, renters, and not married groups had higher BLLs and surface Pb loadings compared to the other categories within these variables. Notable relationships were observed between BLL and surface loading for interior paint damage and household density. Additional associations for surface loading included participant age, structural characteristics, cleaning behaviors, and other sociodemographic variables (e.g., own/rent home, marital status) (Table 2). Surface loading was associated with observation survey ratings of home cleanliness and clutter in the most used room as well as participant perceived neighborhood safety and quality. The drinking water source variable was significant for both BLL and surface wipes, with both tap water and bottled water as the primary consumption having higher geometric mean Pb levels than filtered tap water. The relationship between surface loading and drinking water source is not clear. Participants using filtered water may have had reduced Pb intake from water, but the use of water filters may also represent economic or educational differences among participants.

The NCS results for BLLs were somewhat comparable with pregnant women in NHANES where Mexican Americans were most likely to develop elevated BLL (Wang et al., 2022); in the NCS cohort, the analogous categorization was Hispanic, which may include those who self-identify as Mexican American. A study of pregnant women in Durham, NC also found that Hispanic women had the highest BLL compared to non-Hispanic Black and non-Hispanic White women, which may have been partially attributable to non-U.S. exposure to Pb (Miranda et al., 2010). Geron et al. (2022) found Black/Black-Hispanic and White-Hispanic pregnant women had 35 % and 59.9 % higher urinary Pb levels than White non-Hispanic women, respectively. Asian women in the NCS had the highest geometric mean BLLs and Asians in the 2011–2012 NHANES cycle also had the highest BLLs among reported groups, although results were not reported separately for males and females (CDC, 2022). Another recent evaluation of NHANES in pregnant/breastfeeding participants did not find race as a determinant of BLL, but non-Hispanic Black or other race/ethnicity was positively associated with BLL in non-pregnant women (Bulka et al., 2019). Variables that were predictors of maternal BLL in NHANES included born outside the U.S., alcohol intake, and serum cotinine levels (Bulka et al., 2019). Our one-way ANOVA analysis did not find a similar association for serum cotinine but did find a modest association for pre-pregnancy alcohol consumption (Table 2) with a significant association observed in a multivariable model (Table S-9).

The NCS results for determinants associated with Pb surface loadings were compared with results from other studies. Surface Pb loadings in AHHS II were higher for pre-1960 housing (HUD, 2021), which is consistent with our findings. Similarly, results from the Canadian House Dust Survey (CDHS) showed that higher Pb surface loading rates were associated with older homes (Rasmussen et al., 2013). In logistic regression models for the dust Pb clearance level of 10 μg/ft^2^ (EPA, 2021), the AHHS II variables included were limited to those characterizing Pb-based paint, soil condition, smoking status, and home cleanliness. Of these, the largest predictor of dust Pb loadings was the presence of interior Pb-based paint, irrespective of deterioration; bare soil was also included in the final model (HUD, 2021). The home cleanliness variable was not included in any of these final regression models for dust in the AHHS II study. We did not have soil Pb measurements in the NCS to include in models, however, significant NCS relationships were observed for exterior siding condition and cleanliness of the most used room from the observation survey.

Multivariable Tobit modeling analyses were performed to further assess potential Pb exposure determinants. Table 3 gives multiple regression results for multivariable main effect associations of demographic, behavior/lifestyle, and housing characteristics with BLLs and surface Pb loadings for the pregnant women included in the analyses. Variables included in the main effects models for BLLs and surface Pb loading models were those with one-way ANOVA *p*-values ≤0.10 and ≤ 0.001, respectively. These criteria were chosen to limit the number of variables considered to 15 in the BLL model and 14 in the surface loading model. Many variables may exhibit lesser or greater degrees of co-variability, for example, there are known relationships between race/ethnicity, education, and income in the United States (Shrider et al., 2021). These variables may, in turn, influence other residential and lifestyle characteristics potentially related to Pb exposures. Due to the potential relationships among variables, full models that included interactions were developed in addition to the main effects models.

Main effects model results for BLL and surface Pb loadings indicate only modest overlap for included variables with significant associations (Table 3). For BLL, race/ethnicity, having a full-time job, year home built, interior paint condition, and exterior siding condition remained significant at the α = 0.05 level. Owning or renting the home, home type, household density, and having an attached garage had *p*-values between 0.05 and 0.1. For surface Pb loadings, only the year home built and cleanliness of the most used room in the home remained significant at the α = 0.05 level. Race/ethnicity, interior paint damage, and number of bedrooms had p-values between 0.05 and 0.1, with marital status slightly higher than 0.1. Due to the complicated relationships and potential correlations among variables, coefficient estimates and their p-values may change as variables are added or removed from models (Krzywinski and Altman, 2015).

Full models including interactions give better fits for the data for both blood and surface wipe Pb than the corresponding main effects models based on AIC (Tables S-9 and S-10). These models suggest that the variables found to be significant in the one-way ANOVAs have complicated interrelationships in their associations with BLL and surface wipe Pb, but, beyond that take-away, the models would be difficult to interpret, especially since the NCS cohort is not a representative sample. Variables that are significant in these full models as main effects, in interaction terms, or both for BLL and surface Pb loading are these: race/ethnicity, year home built category, exterior siding condition, interior paint damage, household income, marital status, education, own or rent home, and attached garage. Variables that are significant in the full BLL model but not considered in the surface wipe model are: having a full-time job, pre-pregnancy alcohol consumption frequency, drinking water source, pets in home, home type, and household density. Variables that are significant in the full surface wipe Pb model but not considered in the BLL model are: cleanliness of the most used room and number of bedrooms. The BLL main effects and full models were also fitted using LOD/√2 substitution for non-detects for comparison with the Tobit regression model results (Tables S-11 and S-12); results were generally similar but models using substitution had increased AIC values (poorer fits) and slightly higher p-values.

Many of the variables found to be associated with BLL or surface wipe Pb measurements in this analysis have been observed in previous investigations. Zartarian et al. (2022) described many of the U.S. Pb exposure indices and models developed by federal and state agencies and researchers, which most often include demographic and housing information based on geographic location and U.S. census data. For many indices and models, key information includes race/ethnicity, below poverty level, and housing age; some also include environmental information for Pb in air, soil, and drinking water or information on the presence of Pb-based and deteriorating paint. While the NCS did not collect or report information for some of the variables used in many of the published Pb exposure indices (e.g., environmental Pb levels, measurements of Pb in paint), information for demographic and housing characteristics were available. Information about race/ethnicity is used in most indices and in NHANES was found to be associated with higher BLLs in U.S. women of childbearing age with minority populations having higher BLLs than non-Hispanic White women (Ettinger et al., 2020). Lower household income was associated with higher BLLs and surface loadings in the NCS interaction model, and poverty is a variable used in some Pb exposure indices. Whitehead and Buchanan (2019) discuss the risk of childhood Pb poisoning from an environmental justice perspective, comparing race/ethnicity with other sociodemographic variables. Many of the demographic variables examined in the NCS are related to residential variables, where lower income may lead to living in older or less well-maintained housing that is more likely to have Pb-containing and damaged paint (Isley et al., 2022). Lower-income populations may also experience more crowding in their homes with more soil track-in and wear and tear on home environments (Johnson et al., 2009; Layton and Beamer, 2009). In addition, people living in dense urban neighborhoods typically experience higher soil Pb levels from historically applied Pb-based paint and past fuel-related and industrial activities, potentially leading to higher direct exposures and indoor contamination (Egendorf et al., 2021). Lower-income and minority communities are also more likely to live in closer proximity to industries that produce Pb emissions (Davide et al., 2022). Geographic location is typically a key variable in Pb exposure indices. While the highest NCS BLLs were observed in urban Queens County, NY, the relatively rural Duplin County, NC had the second highest levels. Analysis of child BLL data in South Carolina suggests differences in individual demographics and environmental characteristics may be more closely associated with BLL than geographically aggregated SES and race/ethnicity characteristics (Aelion and Davis, 2019). Housing age is often used in Pb exposure indices and models, and it was found to be associated with both BLLs and surface loadings in the NCS. While direct assessment of the presence of Pb-based paint is likely the best predictor for elevated residential levels of Pb (HUD, 2021), older home age is often used as a surrogate for potential presence of Pb in paint. For example, Dietrich et al. (2022) developed a predictive model for Pb in residential dust that relies only on approximate housing age and whether there is peeling paint in the interior of the home.

The relationship between variables associated with maternal BLL and surface loadings in the NCS may reflect socioeconomic factors, direct physical contact with Pb, or other key determinants not collected. The complexity of the interactions among these potential exposure determinants makes it challenging to tease apart the exact nature of the associations. For example, having a full-time job was associated with lower BLL in the NCS and may be indicative that lower income (from not having a full-time job) may lead to living in older or substandard housing. It may also be that people without full-time jobs spend more time in their home environment resulting in higher exposures to Pb in dust. Having an attached garage was associated with lower BLL and surface loading. The absence of an attached garage may also reflect socioeconomic position; however, the potential for increased track-in of Pb-contaminated soil for those without attached garages cannot be ruled out as a possible exposure source.

Cleanliness of the most used room was strongly associated with Pb surface loading in the NCS. While cleanliness was not associated with Pb surface loadings in the AHHS II final model (HUD, 2021), home cleaning and other interventions have been found to significantly reduce Pb on surfaces in homes; however, reductions do not usually correspond with decreases in child BLL (Braun et al., 2018; Ettinger et al., 2002; Lioy et al., 1998; Nussbaumer-Streit et al., 2020). While the relationship between home surface Pb and cleanliness is not necessarily a novel observation, the strength of the association based on the cleanliness of the most used room rating from the NCS technician-administered observation survey suggests that a cleanliness rating observation may be a useful tool in home Pb assessments.

There are several limitations in this secondary analysis of exposures to Pb in this cohort of pregnant women in the NCS. First, we were unable to include geographic location in the multivariable models. Thus, we were not able to account for observed variability across geographic locations and the potential interactions with other important variables. Second, no NCS Pb measurement data were available for drinking water, foods, or soil which may be important contributors to exposure in some situations. Third, this was a cross-sectional study, not based on a nationally representative sample, that collected BLL measurements at only one point in time during pregnancy; hence, temporal trends and relationships could not be examined. Fourth, only a modest number of children born to these women were followed post-natally with very few urinary Pb measurements over a limited time window of approximately 6 months. Thus, the relationship between maternal and residential Pb and children’s exposures could not be examined with these data.

There are also several strengths in this analysis. While not a nationally representative sample, the NCS IVS included relatively large numbers of pregnant women living in multiple geographic locations of the United States. A wide array of demographic, behavior/lifestyle, and residence information was collected to allow assessment of potential determinants associated with exposures and residential contamination. Finally, the study collected samples for assessing relationships between measures of Pb in the residence and in biological media.

Given Pb’s well established neurological effects resulting from prenatal and early childhood exposures (ATSDR, 2020), it is important to understand maternal exposures during pregnancy and levels of contamination in the residential environments where children will be raised. Data collected in the NCS Vanguard Studies provided valuable concurrent measurements of maternal biomarker and residential surface Pb levels to assess the magnitude and range of exposures for pregnant women in the United States. Extensive survey information, including key demographic, lifestyle, and residential characteristics allowed assessment of potential determinants associated with Pb exposures and residential surface loadings. Although maternal BLLs were all <5 μg/dL, with a central value somewhat below that for non-pregnant women in the general population, widespread exposures are continuing and must be considered in the context of the U.S. CDC’s determination that there is no safe level of exposure to Pb.

The current analysis supports the federal government’s commitment to advancing scientific understanding of Pb exposures and their relationship to BLLs, particularly in an understudied population (President’s Task Force on Environmental Health Risks and Safety Risks to Children, 2018). Further evidence that inequities exist for Pb exposure by pregnant women and young children strengthens the need for public health protections for all lifestages and population groups disproportionately burdened with higher Pb exposures (EPA, 2022). Results from this analysis found associations for Pb levels and race/ethnicity, with higher exposures among minority groups, as well as relationships with other sociodemographic characteristics including lower income. Also observed were associations with home age, a strong indicator of the potential for Pb-based paint, as well as observations of higher Pb loading levels in homes with damaged interior and exterior paint. These demographic and residence determinants have been repeatedly observed in other investigations and reinforces the need for continued efforts to mitigate sources of exposure in low-income and minority communities and for all those living in older housing with Pb paint.

## Supplementary Material

Supplementary Files

## Figures and Tables

**Table 1:** Maternal participant characteristics.

	Participants providing blood samples during pregnancy	Participants with residence surface wipe samples during pregnancy
n	426^a,b^	640^a^
Age at Birth of First NCS Child		
Mean Age	29.2	29.1
Missing or not recorded (%)	6.6	0
Race/Ethnicity (%)		
Hispanic	12.4	13.6
Non-Hispanic White	67.1	64.7
Non-Hispanic Black/African American	5.9	5.3
Non-Hispanic American Indian/Alaskan Native	0	0
Non Hispanic Asian	4.7	3.8
Non-Hispanic Native Hawaiian/Pacific Islander	0.5	0.5
Non-Hispanic multiple races or other race	7.7	8.6
Non-Hispanic missing or other race	1.6	3.6
Household Income (%)		
Less than $30,000	24.4	26.6
$30,000 - $49,999	19.7	16.9
$50,000 - $99,999	28.6	30.6
$100,000 or more	17.4	17.7
Missing/Don’t know	9.9	8.3
Marital Status (%)		
Married	77.2	76.5
Not married/living with partner	22.1	22.8
Never married	0.7	0.5
Divorced, separated, or widowed	0	0.3
Missing	0	0.2
Education (%)		
No high school diploma or GED	24.9	25.5
High school diploma/GED	27.0	28.6
Some college, no degree	38.3	37.7
College degree (AD, BA/BS, post-grad)	9.4	7.8
Missing	0.5	0.5
Trimester Blood Sample Collected (%)		
First	31.2	NA^c^
Second	61.3	NA
Third	0.5	NA
Missing or not recorded	7.0	NA
BMI – Pre-pregnancy (%)		
<18.5	3.5	4.5
18.5–24.9	46.7	47.7
25–29.9	18.3	18.9
≥30	16.9	17.2
Not calculated, insufficient information	14.5	11.7
Cigarette or Other Tobacco Use – Pre-pregnancy (%)		
Yes	14.8	11.6
No	79.3	65.9
Not recorded	5.9	22.5
Alcohol Consumption Frequency - Pre-Pregnancy (%)		
2 or more times a week	12.0	9.7
Once A Week	9.4	7.8
1–3 Times A Month	15.5	12.3
Less Than Once A Month	15.7	15.6
Never	41.8	32.2
Missing or not recorded	5.6	22.3
Year Home Built (%)		
1940 or before	9.9	10.9
1941 to 1960	12.0	11.7
1961 to 1980	23.2	21.7
1981 to 2000	17.1	17.2
2001 to interview date	15.0	17.7
Don’t know	17.8	20.2
Missing or not recorded	4.9	0.6
Pre-Natal Questionnaire Type^d^ (%)		
T1-First	95.1	77.8
T3-First	0.2	21.7
Not available	4.9	0.5

a 357 participants provided both blood and surface wipe samples.

b Three participants excluded due to unresolvable occurrence of two Pb measurements each.

c Not applicable.

d The T1-First questionnaire was generally administered to participants recruited in the first trimester of pregnancy, while the T3-First questionnaire was generally administered to participants recruited during the third trimester. Most questions were identical, but pre-pregnancy tobacco use and alcohol consumption data were not collected in the T3-First questionnaire.

**Table 2:** One-way analysis of variance (ANOVA) results for selected NCS survey variables^a^.

	Mother’s blood			Residential surface wipe		
		GM^c^	Adjusted		GM	Adjusted
Variables/categories	n^b^	μg/dL	p-Value^d^	n^b^	ng/cm^2^	p-Value^d^
Mothers Age at Birth of First NCS Child						
<25	78	0.44	0.3303	138	0.66	**0.0010**
25–29	135	0.41		196	0.46	
30–34	116	0.43		189	0.41	
35–44	69	0.48		117	0.41	
Household Income						
Less than $30,000	104	0.50	**0.0011**	170	0.72	**<0.0001**
$30,000-49,999	84	0.42		108	0.44	
$50,000 - $99,999	122	0.38		196	0.36	
$100,000 or more	74	0.40		113	0.33	
Missing/Don’t know	42	0.57		53	0.76	
Race/Ethnicity						
Hispanic	53	0.58	**<0.0001**	87	0.88	**<0.0001**
Non-Hispanic White	286	0.38		414	0.40	
Non-Hispanic Black/African American	25	0.55		34	0.90	
Non-Hispanic Asian	20	0.99		24	0.49	
Non-Hispanic multiple races	33	0.43		55	0.49	
Non-Hispanic missing or other races	–	–		22	0.31	
Marital Status						
Married	329	0.41	**0.0106**	488	0.40	**<0.0001**
Not married but living together with partner	94	0.51		146	0.82	
Education						
No high school diploma or GED	106	0.58	**<0.0001**	163	0.66	**<0.0001**
High school diploma/GED	115	0.40		183	0.50	
Some college, no degree	163	0.40		241	0.37	
College degree (AD, BA/BS, post-grad)	40	0.39		50	0.34	
wns or Rents Home						
Owned by you/someone in your household	252	0.40	**0.0046**	393	0.39	**<0.0001**
Rented by you/someone in your household	145	0.50		232	0.63	
BMI – Pre-pregnancy						
<18.5	15	0.43	0.9582	29	0.53	**0.0042**
18.5–24.9	199	0.41		305	0.38	
25–29.9	78	0.43		121	0.44	
≥30	72	0.41		110	0.61	
Parity						
0 births or > 24 week stillbirths	30	0.45	0.7042	51	0.60	**0.0423**
1 birth or > 24 week stillbirth	139	0.43		209	0.37	
2 births and/or > 24 week stillbirths	85	0.47		132	0.44	
>2 births and/or > 24 week stillbirths	49	0.41		83	0.48	
Blood Sample Collection Trimester						
First trimester	133	0.43	0.9582	–^e^	–	–
Second trimester	261	0.44		–	–	
Unknown trimester	30	0.44		–	–	
Alcohol Consumption Frequency Pre-Pregnancy						
2–5 Times a week	51	0.44	0.0818	62	0.43	0.1138
Once a week	40	0.37		50	0.37	
1–3 Times a month	66	0.40		79	0.35	
Less than once a month	67	0.40		100	0.43	
Never	178	0.48		206	0.51	
Alcohol Drinks per Day Pre-Pregnancy						
1 per day	121	0.40	0.1273	164	0.37	0.0979
2 per day	67	0.40		83	0.43	
3 per day	21	0.43		26	0.50	
4 or more per day	15	0.45		18	0.33	
Never drink	178	0.48		206	0.51	
Cigarette or Other Tobacco Use Pre-Pregnancy						
Yes	63	0.46	0.6134	74	0.65	**0.0021**
No	338	0.43		422	0.41	
Cigarette or Other Tobacco Use During Pregnancy						
Yes	24	0.53	0.1949	31	0.82	**0.0072**
No	381	0.43		603	0.45	
Environmental Tobacco Smoke Exposure						
Zero hours per day	330	0.43	0.2076	511	0.44	**0.0261**
1 h per day	52	0.45		86	0.55	
2 or more hours per day	20	0.56		34	0.70	
Serum Cotinine						
≤ 0.099 ng/mL	187	0.43	0.9291	309	0.43	**0.0042**
0.137 ng/mL to 3.17 ng/mL	38	0.45		52	0.69	
31.9 ng/mL to 223 ng/mL	16	0.46		28	0.72	
Perceived Stress						
Low	222	0.41	0.1277	355	0.45	0.4799
Moderate or high	179	0.46		270	0.48	
Full-time Job						
No full-time job	203	0.48	**0.0030**	332	0.51	0.0819
One or two full-time jobs	202	0.39		304	0.43	
Number of Jobs						
No full-time or part time or volunteer jobs	119	0.51	**0.0082**	190	0.59	**0.0060**
1 full-time or part time or volunteer jobs	232	0.41		369	0.42	
2 or more full-time or part time jobs	54	0.38		77	0.41	
Handles Chemical(s) on Job						
No chemicals on job	269	0.44	0.9291	424	0.46	0.8428
Never handles chemicals	41	0.44		71	0.49	
Handles chemicals	95	0.42		141	0.48	
Full- or Part-time Student						
Not a student	362	0.44	0.9343	570	0.46	0.2791
Full-time student or part-time student	43	0.43		66	0.55	
Commute Time						
1 to 10 min	138	0.41	0.2076	155	0.40	0.7255
11 to 20 min	96	0.43		128	0.46	
21 to 30 min	67	0.42		81	0.46	
>30 min	62	0.48		76	0.49	
Skip or missing	41	0.52		57	0.41	
Perceived Neighborhood Quality						
Very good place to live	273	0.43	0.9175	437	0.42	**0.0030**
Fairly good place to live	116	0.45		180	0.55	
Not a very good place to live	15	0.44		18	0.87	
Perceived Neighborhood Safety						
Very safe	277	0.42	0.2012	429	0.43	**0.0004**
Somewhat safe	113	0.46		182	0.50	
Somewhat unsafe or very unsafe	14	0.55		24	1.13	
How Long Lived in Home						
≤1 year	126	0.47	0.3651	228	0.47	**0.0193**
>1 to 5 years	216	0.42		319	0.42	
>5 years	63	0.45		90	0.63	
Home Type						
Single family dwelling	264	0.40	**0.0129**	419	0.42	**0.0016**
Duplex	21	0.57		32	0.58	
Multi-family dwelling	22	0.43		36	0.78	
Trailer	30	0.54		53	0.67	
Number of Household Members						
1 or 2	79	0.39	0.2873	123	0.57	**0.0016**
3	119	0.42		182	0.38	
4	97	0.47		154	0.40	
5	51	0.45		80	0.59	
6 or more	59	0.48		98	0.55	
Number of Bedrooms						
0 or 1	19	0.59	**0.0304**	30	1.32	**<0.0001**
2	102	0.48		160	0.56	
3	140	0.42		217	0.41	
4	87	0.41		148	0.40	
5 or more	45	0.36		70	0.36	
Household Density						
≤1.0 person per bedroom	185	0.39	**0.0030**	306	0.40	**0.0013**
1.1–2.0 persons per bedroom	162	0.45		246	0.49	
2.1–3.0 persons per bedroom	32	0.59		43	0.59	
>3.0 persons per bedroom	14	0.54		30	0.88	
Year Home Built						
2001 to Present	64	0.38	**<0.0001**	113	0.22	**<0.0001**
1981 to 2000	73	0.40		110	0.37	
1961 to 1980	99	0.37		139	0.38	
1941 to 1960	51	0.48		75	0.63	
1940 or before	42	0.50		70	1.13	
Don’t know/missing	76	0.58		129	0.71	
Interior Paint Damage						
Interior paint not chipping or peeling	309	0.41	**0.0030**	490	0.39	**<0.0001**
Interior paint chipping or peeling	84	0.53		136	0.84	
NCS Judgement for Interior Paint Risk						
Valid skip for no paint risk	298	0.41	**0.0111**	469	0.38	**<0.0001**
Low paint risk	60	0.49		98	0.73	
Medium or High (blood); Medium (wipe) paint risk	33	0.56		40	0.93	
High paint risk	–	–		16	1.26	
Interior Carpet Quality						
Interior carpet not worn	330	0.43	0.3374	517	0.42	**<0.0001**
Interior carpet worn	63	0.47		109	0.75	
Most Used Room Cleanliness Rating						
Very clean	163	0.41	0.3770	276	0.31	**<0.0001**
Moderately clean	182	0.45		277	0.56	
Not clean	43	0.47		67	1.13	
Most Used Room Clutter Rating						
Little clutter	148	0.43	0.9582	249	0.37	**<0.0001**
Moderately cluttered	156	0.43		246	0.51	
Very cluttered	70	0.44		100	0.70	
Mother Bedroom Cleanliness Rating						
Very clean	163	0.41	0.6062	263	0.32	**<0.0001**
Moderately clean	145	0.44		239	0.53	
Not clean	40	0.42		58	1.04	
Mother Bedroom Clutter Rating						
Little clutter	116	0.42	0.9582	208	0.33	**<0.0001**
Moderately cluttered	153	0.42		223	0.49	
Very cluttered	73	0.43		114	0.65	
Attached Garage						
Yes	174	0.38	**0.0026**	281	0.33	**<0.0001**
No	146	0.48		236	0.66	
Exterior Siding Condition						
Good	218	0.37	**0.0001**	357	0.37	**<0.0001**
Fair	87	0.51		132	0.70	
Poor	20	0.56		29	0.90	
Exterior Paint Chipping						
Exterior paint not chipping	259	0.39	**0.0025**	419	0.38	**<0.0001**
Exterior paint chipping	79	0.52		124	0.92	
Yard Cleanliness						
Little/no debris	219	0.41	0.9343	345	0.40	**0.0001**
Some debris	72	0.42		117	0.58	
Lots of debris	22	0.45		38	0.83	
Home Addition During Pregnancy						
Yes	38	0.44	0.9582	52	0.47	0.9299
No	366	0.43		583	0.46	
Home Projects During Pregnancy						
Yes	100	0.46	0.4987	173	0.49	0.5510
No	304	0.43		462	0.46	
Drinking Water Source						
Tap water	122	0.44	**0.0423**	179	0.53	**0.0011**
Filtered tap water	154	0.39		243	0.37	
Bottled water	116	0.49		196	0.56	
Some other source	–	–		17	0.38	
Cooking Water Source						
Tap water	341	0.43	0.5666	535	0.46	0.7144
Filtered tap water	39	0.49		63	0.53	
Bottled water	18	0.44		26	0.54	
Some other source	–	–		10	0.40	
Pets in Home						
Yes	207	0.40	**0.0219**	316	0.48	0.7113
No	196	0.47		319	0.46	

a ANOVA test results significant at the *p* < 0.05 level are shown in bold.

b The number of observations may be less than the total measurements as categories or missing results with <10 observations were not included in the ANOVA analysis; in some cases categories were combined for analysis. Participants not administered survey instruments were not included in the ANOVA analysis.

c GM is the geometric mean of Pb measurements; non-detect values for BLLs were substituted with the LOD/√2.

d Benjamini-Hochberg multiple testing adjustment method.

e ANOVA analysis not performed for this variable for surface wipe measurements.

**Table 3:** Multivariable model assessment of potential exposure predictors for Pb – main effects multiple regression model results.

	Mother’s Blood^a^		Residential surface wipe^b^	
Number of observations	283		450	
Number of censored observations	48		0	
AIC	496.4		1253.3	
Effect	Wald Chi-square	Pr > ChiSq	F value	Pr > F
Household Income	4.81	0.3070	0.49	0.7451
Race/Ethnicity	19.23	**0.0007**	2.18	0.0560
Marital Status	0.52	0.4698	2.68	0.1022
Education	2.73	0.4351	0.47	0.7051
Alcohol Consumption Frequency Pre-Pregnancy	7.30	0.1211	NI^c^	NI
Full-time Job	9.70	**0.0018**	NI	NI
Own or Rents Home	3.62	0.0572	0.05	0.8302
Home Type	7.03	0.0710	NI	NI
Household Density	6.41	0.0934	NI	NI
Year Home Built	11.49	**0.0425**	12.72	**<0.0001**
Interior Paint Damage	5.51	**0.0189**	3.71	0.0549
Attached Garage	2.70	0.1007	0.05	0.8151
Exterior Siding Condition	11.61	**0.0030**	0.53	0.5866
Drinking Water Source	1.65	0.4388	NI	NI
Pets in Home	0.14	0.7120	NI	NI
Perceived Neighborhood Safety	NI	NI	0.46	0.6326
Number of Bedrooms	NI	NI	2.08	0.0823
Interior Carpet Quality	NI	NI	1.45	0.2291
Most Used Room Cleanliness Rating	NI	NI	12.74	**<0.0001**
Yard Cleanliness	NI	NI	0.21	0.8133

a Modeled using Tobit regression in the SAS LIFEREG procedure due to <LOD Pb measurement values.

b Modeled using multiple regression in the SAS MIXED procedure; no Pb measurements were <LOD.

c NI = not included in the model.

## Data Availability

This manuscript was prepared using National Children’s Study Research Materials obtained from the NCS Vanguard Data and Sample Archive and Access System and the NICHD Data and Specimen Hub (DASH). We acknowledge NICHD DASH for providing the National Children’s Study data that was used for this research. We appreciate the contributions of the NCS NICHD Principal Investigator Jack Moye, and the NCS Initial Vanguard Center Principal Investigators: Children’s Hospital of Philadelphia (Jennifer Culhane); Mt. Sinai Medical School (Philip Landrigan); South Dakota State University (Bonny Specker); University of California at Irvine (James Swanson and Dean Baker); University of North Carolina at Chapel Hill (Barbara Entwisle and Nancy Dole); University of Utah School of Medicine (Ed Clark); and the University of Wisconsin (Maureen Durkin). Data are not publicly available, as they were obtained through Data Use Agreements.

## HIGHLIGHTS

- Secondary data analysis of a 2009–2014 U.S. cohort of pregnant women
- Blood and urine biomarker measurements provide evidence of Pb exposures.
- Blood Pb levels are significantly correlated with surface wipe Pb measurements.
- Demographic and residential characteristics are associated with Pb measurements.
- Higher Pb exposures for minority and lower-income women and living in older homes
