# Supplementary material for: Assessing lead exposure in U.S. pregnant women using biological and residential measurements
Source: Sci Total Environ. Author manuscript; Available in PMC 2024 Dec 20. (PMC11351066; doi:10.1016/j.scitotenv.2023.167135)
Supplement: Supplementary Files [file NIHMS2017723-supplement-Supplementary_Files.docx]

**Supplemental Information for:**

**Assessing Lead Exposure in U.S. Pregnant Women using Biological and Residential Measurements**

Lindsay W. Stanek^a,*^, Nicholas Grokhowsky^b^, Barbara J. George^a^, Kent W. Thomas^a^

^a^ U.S. Environmental Protection Agency (EPA), Office of Research and Development (ORD), Research Triangle Park, North Carolina, USA

^b^ Formerly of Oak Ridge Institute for Science and Education, Research Triangle Park, North Carolina, USA

**Table S-1. Criteria used to select participant IDs included in Pb study**

| Media | Blood | Surface Wipe |
| --- | --- | --- |
| NCS file (SAS7BDAT format) | vs_bloodlab_results_v2 | IVS_WIPE_METALS_V1 |
| Count of observations | 44796 | 21525 |
| Observations included for variable names and their values | |  |
| Participanttype | 2 (Mother) | 2 (Mother) |
| Eventtype | T1-MOTHER | 4 (Pregnancy Visit 1 – First Trimester), |
|  |  | 5 (Pregnancy Visit 1 – Third Trimester) |
| Matrix variable name and the values | |  |
| Materialtype | Whole Blood | Wipe |
| Count of observations for Pb after removing observations for duplicate IDs | 3408 | 16000 |
| Number of distinct participant IDs with a single Pb measurement | 426 | 640 |
| Number of IDs with Pb results ≥LOD | 366 | 640 |
| Number <LOD | 60 | 0 |

**Table S-2. Information for New Variable Derivation**

| **Derived Variable Categories** | **Coding Specifications Using DASH^a^ NCS Variable Names** | |
| --- | --- | --- |
| Tobacco User – 3 Months Pre-Pregnancy | |  |
| 1=Yes | coded as 1=Yes if either CIGARETTEUSEPREPREG=1 or OTHTOBACCOUSEPREPREG=1 (or both) | |
| 2=No | else coded as 2=No if either CIGARETTEUSEPREPREG=2 or OTHTOBACCOUSEPREPREG=2 (or both) | |
| Tobacco User – Current (during pregnancy) | |  |
| 1 = Yes | coded as 1=Yes if either CIGARETTEUSECURRENT=1 or OTHTOBACCOUSECURRENT=1 (or both) | |
| 2 = No | else coded as 2=No if either CIGARETTEUSECURRENT=2 or OTHTOBACCOUSECURRENT=2 (or both) | |
| Perceived Stress (From 10-item Perceived Stress Scale In Last Month)^b^ | |  |
| 0-13 Low Perceived Stress | recode CPSSUPSETUNEXPECTEDLY, CPSSUNABLETOCONTROL, CPSSNERVOUSSTRESSED, CPSSCOPETHINGSTODO, CPSSANGEREDNOCONTROL, CPSSDIFFICULTIESHIGH | to 0=Never, 1=Almost Never, 2=Sometimes, 3=Fairly Often, 4=Very Often |
| 14-26 Moderate Perceived Stress | recode CPSSCONFIDENTABILITY, CPSSTHINGSYOURWAY, CPSSCONTROLIRRITATIONS, CPSSONTOPOFTHINGS | to 4=Never, 3=Almost Never, 2=Sometimes, 1=Fairly Often, 0=Very Often |
| 27-40 High Perceived Stress | sum the recoded values for the 10 variables and group into categories |  |
| Household Person Density |  |  |
| 1 = 0-1.0 | categories based on TOTALNUMBERHHMEMBERS / BEDRMS | |
| 2 = 1.1-2.0 |  |  |
| 3 = 2.1-3.0 |  |  |
| 4 = >3.0 |  |  |
| How Long Participant Lived in Current Home | |  |
| 1 = ≤1 year | categories based on |  |
| 2 = >1 to ≤5 years | HOWLONGLIVEDINHOME if HOWLONGLIVEDINHOMEUNIT=3 |  |
| 3 = >5 years | HOWLONGLIVEDINHOME/12 if HOWLONGLIVEDINHOMEUNIT=2 |  |
|  | HOWLONGLIVEDINHOME/52 if HOWLONGLIVEDINHOMEUNIT=1 |  |
| Parity – Number of prior live births and stillborn >24weeks | |  |
| 1 = 0 births or >24 week stillbirths | categories based sum of NUMBEROFLIVEBIRTHS and NUMBERSTILLBIRTHS | |
| 2 = 1 births or >24 week stillbirth |  |  |
| 3 = 2 births and/or >24 week stillbirths |  |  |
| 4 = >2 births and/or >24 week stillbirths |  |  |
| BMI Pre-Pregnancy |  |  |
| 1 = <18.5 | categorize BMI = weight (lb) / [height (in)]^2^ x 703 (CDC formula for BMI) based on |  |
| 2 = 18.5 – 24.9 | total height (in) = 12*PREPREGHEIGHTFEET + PREPREGHEIGHTINCHES |  |
| 3 = 25 – 29.9 | weight (lb) = 2.205 * PREPREGWEIGHTKILOS, if weight reported in kilos, |  |
| 4 = ≥30 | or weight (lb) = PREPREGWEIGHTLBS, if weight reported in pounds |  |

^a^ NICHD Data and Specimen Hub; Moye, 2020.

^b^ Based on the 10-item Perceived Stress Scale (PSS-10) (Cohen et al., 1983; Cohen and Williamson, 1988)

**Table S-3. Summary statistics for NCS Pb measurements**

|  | Whole  Blood | Urine (unadjusted) | Urine  (creatinine adjusted) | Surface  Wipes (during pregnancy) | Surface Wipes  (6-month post-partum) | House  Dust |
| --- | --- | --- | --- | --- | --- | --- |
| n | 426 | 366 | 366 | 640 | 99 | 208 |
| % >LOD | 86 | 97 | 97 | 100 | 100 | 100 |
|  |  |  |  |  |  |  |
| *Units* | µg/dL | µg/L | µg/g | ng/cm^2^ | ng/cm^2^ | mg/kg |
| LOD | 0.25 | 0.08 | 0.11 | 0.011 – 1.1 | 0.011 – 1.1 | 0.45 – 11 |
| Geometric Mean ^a^ | 0.44 | 0.36 | 0.43 | 0.47 | 0.49 | 37 |
| 95^th^ CI for Geometric Mean ^a^ | 0.41 – 0.46 | 0.33 – 0.39 | 0.41 – 0.46 | 0.43 – 0.51 | 0.39 – 0.61 | 32 – 43 |
| Min | <LOD | <LOD | <LOD | 0.021 | 0.066 | 2.3 |
| 10^th^ Percentile | <LOD | 0.13 | 0.22 | 0.12 | 0.17 | 13 |
| 25^th^ Percentile | 0.31 | 0.22 | 0.29 | 0.20 | 0.26 | 17 |
| 50^th^ Percentile | 0.42 | 0.36 | 0.41 | 0.42 | 0.39 | 28 |
| 75^th^ Percentile | 0.60 | 0.62 | 0.58 | 0.93 | 0.85 | 60 |
| 90^th^ Percentile | 0.92 | 1.1 | 0.95 | 2.2 | 1.6 | 190 |
| 95^th^ Percentile | 1.3 | 1.4 | 1.3 | 3.6 | 2.8 | 510 |
| Max | 3.1 | 4.7 | 3.8 | 72 | 68 | 1350 |

^a^Censoring-adjusted maximum likelihood estimate (MLE) using R package *EnvStats*

**Table S-4. NCS blood Pb comparison with NHANES**

|  | **NCS**  **(2009-2010)** | **NHANES Pregnant Women**  **(2011-2016)^a^** | **NHANES Non-pregnant Women**  **(2011-2016)^a^** | **NHANES**  **Females**  **(2009-2010)^b,c^** |
| --- | --- | --- | --- | --- |
| n | 426 | 103 | 2181 | 4427 |
| Geometric Mean (95% CI) | 0.44 (0.41,0.46) | 0.48  (0.39,0.57) | 0.61  (0.59,0.64) | 0.967  (0.93, 1.01) |
| BLL % ≥5 µg/dL  [% (95% CI)] | 0.0 | 2.8  (0.0,11.0) | 0.7  (0.3,1.2) | Not reported |

^a^From Ettinger et al., 2020

^b^From Centers for Disease Control and Prevention, National Health and Nutrition Examination Survey (NHANES) National Exposure Report (2022)

^c^Participants - 6+ years of age

**Table S-5. Differences in BLL and Surface Wipe Pb Across NCS Study Locations**

|  | Blood Pb (µg/dL) | | |  | Surface Wipe Pb (ng/cm^2^) | | |
| --- | --- | --- | --- | --- | --- | --- | --- |
| Location | n | GM^a^ | GSD^b^ |  | n | GM^a^ | GSD^b^ |
| Duplin County, NC | 45 | 0.54 | 1.77 |  | 96 | 0.82 | 2.48 |
| BYPL, SD/MN^c^ | 100 | 0.35 | 1.74 |  | 145 | 0.33 | 3.29 |
| Montgomery County, PA | 61 | 0.45 | 1.80 |  | 67 | 0.65 | 3.91 |
| Orange County, CA | 51 | 0.50 | 1.86 |  | 80 | 0.40 | 2.66 |
| New York City (Queens), NY | 36 | 0.72 | 1.88 |  | 46 | 1.21 | 2.90 |
| Salt Lake County, UT | 100 | 0.37 | 1.71 |  | 120 | 0.41 | 2.67 |
| Waukesha County, WI | 28 | 0.39 | 1.37 |  | 86 | 0.31 | 2.60 |
| Overall | 421 | 0.43 | 1.81 |  | 640 | 0.47 | 3.15 |
|  |  |  |  |  |  |  |  |
| F-Statistic |  | 10.3 |  |  |  | 17.0 |  |
| p-value for F test |  | <0.0001 |  |  |  | <0.0001 |  |
|  |  |  |  |  |  |  |  |

^a^GM = geometric mean

^b^GSD = geometric standard deviation

^c^BYPL = Brookings County, SD; Yellow Medicine, Lincoln, Pipestone Counties, MN

**Table S-6. Blood lead levels measured during pregnancy in selected cohorts from multiple countries**

| Country | Year | Maternal Age (Mean) | Blood Collection Time | n | LOD | % <LOD | units^a^ | Median | Geometric Mean | Min | Max | Citation |
| --- | --- | --- | --- | --- | --- | --- | --- | --- | --- | --- | --- | --- |
| U.S. (this study) | 2009-2010 | 29.2 | 6 - 32 weeks | 426 | 0.25 | 14 | μg/dL | 0.42 | 0.44 | ND | 3.1 | This study |
| Canada | 2008-2011 | 32.2 | 6 - 13 weeks | 1938 | 0.10 | 0 | μg/dL | 0.60 | 0.62 | 0.15 | 5.18 | Arebuckle (2016) |
| Canada | 2008-2011 | 32.2 | 32 - 34 weeks | 1673 | 0.10 | 0.18 | μg/dL | 0.56 | 0.57 | ND | 4.14 | Arebuckle (2016) |
| Mexico | 2007-2012 | 27.1 | 2nd trimester | 487 | <0.2 | 0 | μg/dL | nr^b^ | 3.0 | 0.8 | 17.8 | Renzetti (2017) |
| Mexico | 2007-2013 | 27.1 | 3rd trimester | 428 | <0.2 | 0 | μg/dL | nr | 3.1 | 0.3 | 28.3 | Renzetti (2017) |
| Grenada | 2008-2011 | 26.5 | pregnancy or delivery | 52 | 0.52 | 0 | μg/dL | 1.09 | 1.17 | nr | 4.14 | Forde (2014) |
| St. Vincent & the Grenadines | 2008-2011 | 26.7 | pregnancy or delivery | 50 | 0.52 | 2 | μg/dL | 2.07 | 1.98 | nr | 7.87 | Forde (2014) |
| Brazil | 2019-2022 | 27.0 | 12 - 24 weeks | 117 | 0.1 | nr | μg/dL | 0.90 | nr | nr | nr | Bah (2023) |
| Spain | 2016-2017 | 34.0 | 1st trimester | 48 | 0.05 | 0 | μg/dL | 1.0 | nr | 0.38 | 5.2 | Bocca (2019) |
| Spain | 2016-2017 | 34.0 | delivery | 40 | 0.05 | 0 | μg/dL | 1.2 | nr | 0.52 | 4.1 | Bocca (2019) |
| Spain | 2003-2004 | 31.1 | pregnancy | 140 | 0.17 | 0 | μg/dL | nr | 1.98 | nr | nr | Garcia-Esquinas (2013) |
| Norway | 2002-2008 | nr | week 18 | 2982 | nr | 0 | μg/dL | 0.82 | 0.83 | ND | 21.2 | Caspersen (2019) |
| Poland | 2014-2015 | 28.1 | delivery | 136 | 0.28 | 8.5 | μg/dL | 1 | nr | ND | 14 | Kot (2021) |
| China | 2010-2012 | 27.0 | 1st, 2nd, 3rd trimester | 1400 | 0.002^d^ | 0.3 | μg/dL | 0.19^c^ | nr | nr | nr | Liu (2017) |
| Japan | 2011-2014 | 31.2 | Mean 27 weeks (IQR 25-29) | 17,997 | [0.13]^e^ | 0 | μg/dL | 0.63 | 0.64 | 0.16 | 7.45 | Nakayama (2019) |
| Australia | 2008-2011 | 32.0 | 2 weeks before delivery | 173 | 0.05 | 11 | μg/dL | 0.37 | nr | ND | 2.55 | Hinwood (2013) |

^a^Units converted to μg/dL if reported in different units in the cited study

^b^nr = not reported in the study journal article

^c^Pb measurements made in serum instead of whole blood

^d^This study reported a limit of quantitation (LOQ)

^e^LOD reported in units of ng/g

**Table S-7. NCS Creatinine-adjusted Urinary Pb comparison with NHANES^a^**

|  | **NCS (µg/g)**  **2009-2010** | **NHANES (µg/g)  Age 20+ years**  **2009-2010^a^** | **NHANES (µg/g) Females**  **2009-2010 ^a,b^** | **NHANES (µg/g) Age 20+ years**  **2011-2012 ^a^** | **NHANES (µg/g) Females**  **2011-2012 ^a,b^** |
| --- | --- | --- | --- | --- | --- |
| n | 366 | 2019 | 1450 | 1714 | 1241 |
| Geometric Mean (95% CI) | 0.43 (0.41,0.46) | 0.51 (0.49,0.54) | 0.49 (0.47,0.53) | 0.43 (0.40,0.47) | 0.43 (0.40,0.46) |
| 90^th^ Percentile (95% CI) | 0.95^c^ | 1.22 (1.10,1.35) | 1.20 (1.08,1.32) | 1.05 (0.929,1.22) | 1.03 (0.905,1.21) |

^a^From Centers for Disease Control and Prevention, National Health and Nutrition Examination Survey (NHANES) National Exposure Report (2022)

^b^Participants - 6+ years of age

^c^CI not calculated

**Table S-8. NCS Residential Surface Wipe Pb Comparison with AHHS**

|  | **NCS (ng/cm^2^)**  **2009-2010** | **AHHS I^a^ (ng/cm^2^)**  **2005-2006** | **AHHS II^a^ (ng/cm^2^)**  **2018-2019** |
| --- | --- | --- | --- |
| n | 640 | 1131 | 703 |
| Arithmetic Mean (95% CI) | 1.23^c^ | 3.83  (2.64,5.04) | 3.96  (2.25,5.67) |
| Median (95% CI) | 0.47  (0.43,0.51) | 0.61  (0.52,0.70) | 0.33  (0.26,0.40) |
| 90^th^ Percentile (95% CI) | 2.2^c^ | 5.29  (4.14,7.65) | 5.27  (3.56,7.23) |

^a^From Table 6-3 and Appendix C in U.S. Department of Housing and Urban Development (2021)

^b^Geometric mean

^c^CI not calculated

**Table S-9. Multivariable model assessment of potential exposure predictors for mother’s blood Pb – full multiple regression model results^a^**

|  | **Mother’s Blood** | |
| --- | --- | --- |
| Number of Observations | 283 | |
| Number of Left-Censored Observations | 48 | |
| AIC | 437.9 | |
|  |  |  |
| **Effect** | **Wald Chi-square** | **Pr > ChiSq** |
| Household Income | 25.43 | **<.0001** |
| Race/Ethnicity | 4.75 | 0.3135 |
| Household Income * Race/Ethnicity | 51.22 | **<.0001** |
| Race/Ethnicity * Home Type | 52.55 | **<.0001** |
| Marital Status | 0.68 | 0.4101 |
| Household Income * Marital Status | 10.78 | **0.0291** |
| Education | 10.89 | **0.0124** |
| Alcohol Consumption Frequency Pre-Pregnancy | 18.10 | **0.0012** |
| Full-time Job | 5.12 | **0.0236** |
| Own or Rents Home | 0.00 | 0.9998 |
| Own or Rents Home * Home Type | 6.48 | **0.0391** |
| Home Type | 20.46 | **0.0001** |
| Household Density | 9.32 | **0.0253** |
| Year Home Built | 43.66 | **<.0001** |
| Household Income * Year Home Built | 42.03 | **0.0004** |
| Year Home Built * Home Type | 61.75 | **<.0001** |
| Interior Paint Damage | 1.47 | 0.2253 |
| Year Home Built * Interior Paint Damage | 8.65 | 0.1238 |
| Attached Garage | 20.29 | **<.0001** |
| Year Home Built * Attached Garage | 28.12 | **<.0001** |
| Exterior Siding Condition | 7.23 | **0.0269** |
| Year Home Built * Exterior Siding Condition | 29.58 | **0.0005** |
| Exterior Siding Condition * Interior Paint Damage | 3.90 | 0.1425 |
| Year Home Built * Exterior Siding Condition * Interior Paint Damage | 22.23 | **0.0011** |
| Drinking Water Source | 4.93 | 0.0851 |
| Alcohol Consumption Frequency * Drinking Water Source | 21.36 | **0.0063** |
| Pets in Home | 0.00 | 0.9853 |
| Household Income * Pets in Home | 12.13 | **0.0164** |
| Pets in Home * Household Density | 9.95 | **0.0069** |

^a^Modeled using Tobit regression in the SAS LIFEREG procedure due <LOD Pb measurement values

**Table S-10. Multivariable model assessment of potential exposure predictors for residential surface wipe Pb – full multiple regression model results^a^**

|  | **Residential Surface Wipe^b^** | |
| --- | --- | --- |
| Number of Observations | 450 | |
| AIC | 1191.6 | |
|  |  |  |
| **Effect** | **F Value** | **Pr > F** |
| Household Income | 1.91 | 0.1082 |
| Race/Ethnicity | 2.99 | **0.0117** |
| Marital Status | 0.68 | 0.4118 |
| Education | 2.31 | 0.0763 |
| Race/Ethnicity * Education | 3.37 | **0.0002** |
| Own or Rents Home | 0.21 | 0.6447 |
| Number of Bedrooms | 3.57 | **0.0071** |
| Marital Status * Number of Bedrooms | 3.36 | **0.0102** |
| Own Or Rents Home * Number of Bedrooms | 2.39 | 0.0503 |
| Year Home Built | 1.58 | 0.1642 |
| Year Home Built * Own Or Rents Home | 3.61 | 0.0033 |
| Interior Paint Damage | 0.13 | 0.7196 |
| Household Income * Interior Paint Damage | 4.51 | **0.0014** |
| Attached Garage | 1.14 | 0.2871 |
| Attached Garage * Interior Paint Damage | 8.90 | **0.0030** |
| Exterior Siding Condition | 0.59 | 0.5552 |
| Year Home Built * Exterior Siding Condition | 0.93 | 0.4965 |
| Own Or Rents Home * Exterior Siding Condition | 5.55 | **0.0042** |
| Year Home Built * Own Or Rents Home * Exterior Siding Condition | 2.56 | **0.0136** |
| Most Used Room Cleanliness Rating | 20.04 | **<.0001** |

^a^Modeled using SAS MIXED procedure; no Pb measurements were <LOD

**Table S-11. Multivariable model assessment of potential exposure predictors for Pb – main effects multiple regression blood model results using LOD/√2 substitution in SAS MIXED procedure**

|  | **Mother’s Blood** | |
| --- | --- | --- |
| Number of Observations | 283 | |
| Number of Observations with Substitutions | 48 | |
| AIC | 503.2 | |
|  |  |  |
| Effect | F-Value | Pr > F |
| Household Income | 1.03 | 0.3925 |
| Race/Ethnicity | 4.42 | **0.0018** |
| Marital Status | 0.4 | 0.5278 |
| Education | 0.81 | 0.4886 |
| Alcohol Consumption Frequency Pre-Pregnancy | 1.74 | 0.1428 |
| Full-time Job | 8.53 | **0.0038** |
| Own or Rents Home | 3.46 | 0.0639 |
| Home Type | 2.11 | 0.0995 |
| Household Density | 1.95 | 0.1225 |
| Year Home Built | 2.04 | 0.0738 |
| Interior Paint Damage | 4.81 | **0.0292** |
| Attached Garage | 2.44 | 0.1193 |
| Exterior Siding Condition | 5.13 | **0.0066** |
| Drinking Water Source | 0.79 | 0.4555 |
| Pets in Home | 0.13 | 0.7167 |

**Table S-12. Multivariable model assessment of potential exposure predictors for mother’s blood Pb – full model results using LOD/√2 substitution in SAS MIXED procedure**

|  | Mother’s Blood | |
| --- | --- | --- |
| Number of Observations | 283 | |
| Number of Observations with Substitutions | 48 | |
| AIC | 359.2 | |
| Effect | F-Value | Pr > F |
| Household Income | 3.31 | **0.0123** |
| Race/Ethnicity | 0.46 | **0.7668** |
| Household Income * Race/Ethnicity | 3.04 | **0.0022** |
| Race/Ethnicity * Home Type | 3.89 | **0.0006** |
| Marital Status | 0.32 | 0.5730 |
| Household Income * Marital Status | 1.38 | 0.2422 |
| Education | 1.69 | 0.1713 |
| Alcohol Consumption Frequency Pre-Pregnancy | 2.48 | **0.0460** |
| Full-time Job | 2.23 | 0.1374 |
| Own or Rents Home | 0.13 | 0.7185 |
| Own or Rents Home * Home Type | 2.28 | 0.1061 |
| Home Type | 3.51 | **0.0168** |
| Household Density | 1.23 | 0.2992 |
| Year Home Built | 4.26 | **0.0012** |
| Household Income * Year Home Built | 1.35 | 0.1713 |
| Year Home Built * Home Type | 3.11 | **0.0008** |
| Interior Paint Damage | 0.51 | **0.4766** |
| Year Home Built * Interior Paint Damage | 0.99 | **0.4243** |
| Attached Garage | 10.82 | **0.0012** |
| Year Home Built * Attached Garage | 3.04 | **0.0119** |
| Exterior Siding Condition | 2.11 | **0.1245** |
| Year Home Built * Exterior Siding Condition | 1.64 | **0.1074** |
| Exterior Siding Condition * Interior Paint Damage | 1.03 | **0.3610** |
| Year Home Built * Exterior Siding Condition * Interior Paint Damage | 2.3 | **0.0376** |
| Drinking Water Source | 1.4 | 0.2501 |
| Alcohol Consumption Frequency * Drinking Water Source | 1.34 | 0.2278 |
| Pets in Home | 0.86 | 0.3553 |
| Household Income * Pets in Home | 1.75 | 0.1417 |
| Pets in Home * Household Density | 2.56 | 0.0809 |


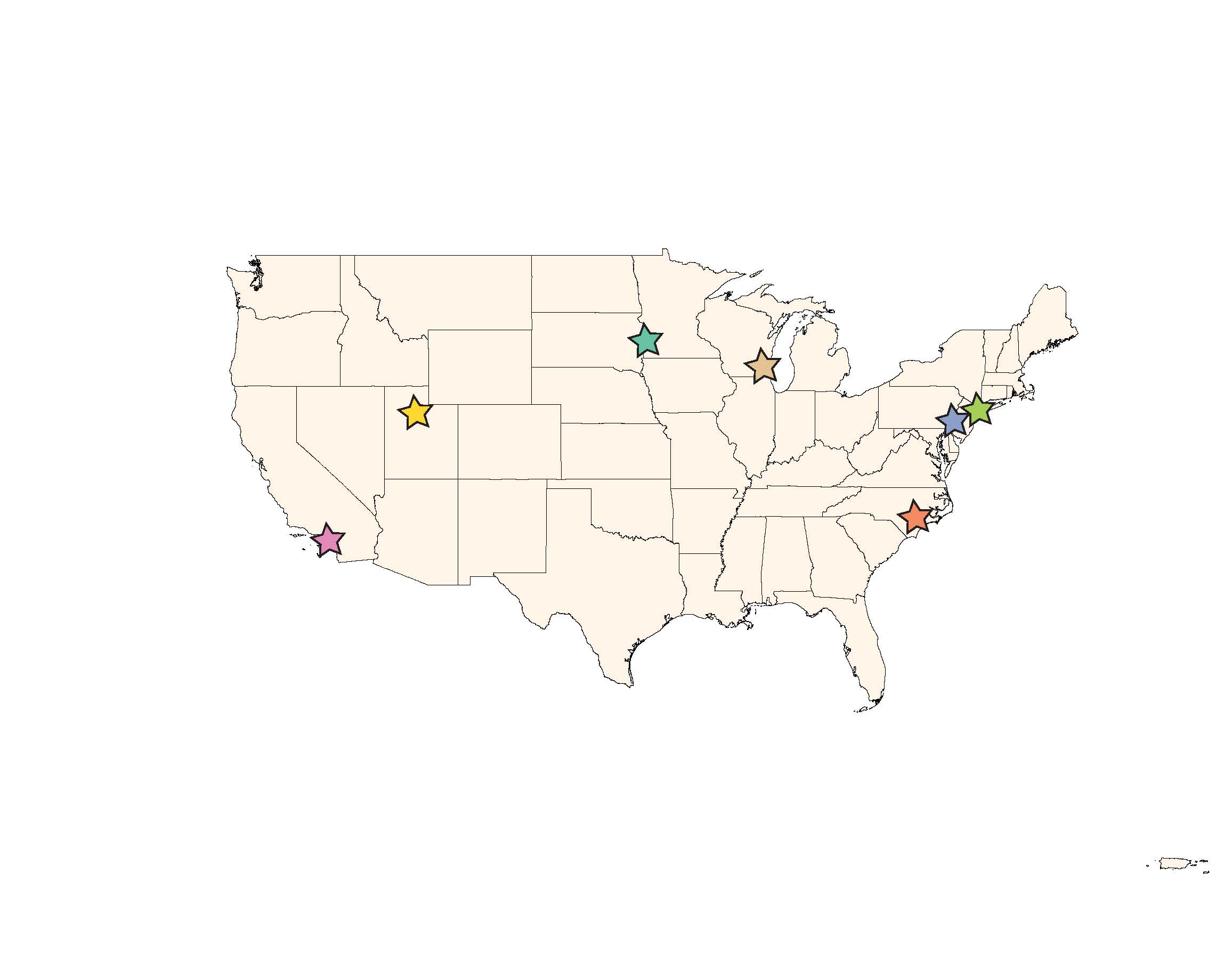


Salt Lake County, UT (n=100)

Orange County, CA (n=51)

Waukesha County, WI (n=28)

Montgomery County, PA (n=61)

Brookings County, SD; Yellow Medicine, Lincoln, Pipestone Counties, MN (n=100)

Duplin County, NC (n=45)

Queens County, NY (n=36)

**Figure S-1. Geographic locations of NCS Initial Vanguard Study counties.**
